# Unfolding the innovation system for the development of countries: coevolution of Science, Technology and Production

**DOI:** 10.1038/s41598-019-52767-5

**Published:** 2019-11-11

**Authors:** Emanuele Pugliese, Giulio Cimini, Aurelio Patelli, Andrea Zaccaria, Luciano Pietronero, Andrea Gabrielli

**Affiliations:** 1 Istituto dei Sistemi Complessi (ISC)-CNR, 00185 Rome, Italy; 2 International Finance Corporation, World Bank Group, 20433 Washington, USA; 3 University of Bath, Bath, BA27AY United Kingdom; 4 IMT School for Advanced Studies, 55100 Lucca, Italy; 5 Dipartimento di Fisica, Sapienza Università di Roma, 00185 Rome, Italy; 6 Present Address: European Commission, Joint Research Centre (JRC), 41092 Seville, Spain; 7 Present Address: Service de Physique de l’Etat Condensé, CEA-Saclay, 91191 Gif-sur-Yvette, France; 8 Present Address: Dipartimento di Ingegneria, Università Roma 3, 00146 Rome, Italy

**Keywords:** Computational science, Complex networks

## Abstract

We show that the space in which scientific, technological and economic activities interplay with each other can be mathematically shaped using techniques from statistical physics of networks. We build a holistic view of the innovation system as the tri-layered network of interactions among these many activities (scientific publication, patenting, and industrial production in different sectors), also taking into account the possible time delays. Within this construction we can identify which capabilities and prerequisites are needed to be competitive in a given activity, and even measure how much time is needed to transform, for instance, the technological know-how into economic wealth and scientific innovation, being able to make predictions with a very long time horizon. We find empirical evidence that, at the aggregate scale, technology is the best predictor for industrial and scientific production over the upcoming decades.

## Introduction

Knowledge production and organization represents the main activity of modern societies – “learning economies”^1^ in which most of the wealth of a country is intangible, and the organization of the national innovation system^2^, and of diffused creativity^3^ are the crucial capabilities for success. Therefore, in the last thirty years the relationships between science, technology and economic competitiveness have become an important focus for social sciences in general and economics in particular^4,5^. Even though the layman narrative links science, technology and economic productivity in a direct flow^6^, actual interactions on the fine-grained scale of individual activities are typically multi-directional and far more entangled^7,8^. The literature on industrial organization and evolutionary economics changed the description of the innovation system from a directed chain to a co-evolution of distinct processes driven by different motives and routines^9^. This non-hierarchical and multi-directional interplay among the individual components of the innovation system is indeed the footprint of a complex system.

Differently from the traditional social science approach, the new techniques of Economic Complexity do not try to average out the complexity of the system, but embrace it by explicitly building on the heterogeneity of individual actors, activities and interactions to extract the relevant and statistically robust parameters characterizing the system. Trying to recover the qualitative insights^10^ and the few quantitative attempts^11,12^ of the heterodox economists and social scientists, researchers used this approach to study unobservable features and capabilities of countries^13–15^, and to unearth unexpected synergies among different activities^16,17^ (see also^18^).

Following this line^11,16,17,19^, here we create the network of interactions between the different human activities involved in the innovation system. Differently from previous analyses, that describe the interactions between very general and broad activities (*e.g*., science and technology) or represent specific case studies (*e.g*., relationships between technological development and productivity in the semiconductor industries), our construction provides, for the first time, a *disaggregated* picture of the bidirectional impact between *all* the possible pairs of activities (*e.g*., export of semiconductor transistors and technological development in organic chemistry), using a uniform methodology with a few controlled assumptions. In particular, our network encompasses activities in different realms (or *layers*): scientific fields, technological sectors, and economic production. We build such a comprehensive *multi-layer* network^20^, namely a system where entities belong to different sets and several categories of connections exist among them, using the following key assumption: if two activities co-occur significantly more often than randomly (in terms of appropriate null models) in the same countries at given times, then there is an overlap between the capabilities required to achieve proficient level (*i.e*., competitive advantage) in both. Notice that in this narrative we use a very wide definition of the term capability, to mean at the same time physical and intangible (cultural or technological) resources. In particular, the presence of an activity could be intended itself as a capability for a different activity (for example through complementarities and spillovers^21^).

As detailed in the Methods section, the starting point of our construction are the bi-adjacency matrices *M*_*c*,*a*_^*L*^(*y*) whose elements indicate whether country *c* has a competitive advantage in activity *a* belonging to layer *L*, in year *y*. *L* stands for the layer of analysis, consisting in the set of all activities related to either *S*cience, *T*echnology or *P*roducts export. Note that each of these layers has an intrinsic hierarchical structure: for instance, in the science layer we can consider activities like *Physics and Astronomy* or corresponding sub-activities (like *Statistical Physics, Condensed Matter Physics, Nuclear and High Energy Physics*). Thus, our matrices do depend on the resolution used for activities classification (even if not explicitly reported in the notation). We use different established databases to construct the multi-layer space: for *S*cience, we take bibliometric data on papers in the various scientific fields from Scopus (www.scopus.com); for *T*echnology, we consider the number of patents in different technological sectors extracted from Patstat (www.epo.org/searching-for-patents/business/patstat); and for *P*roducts, we use export data collected by UN COMTRADE (https://comtrade.un.org/)—which are typically used as proxy of a competitive industrial production.

**Figure 1:**
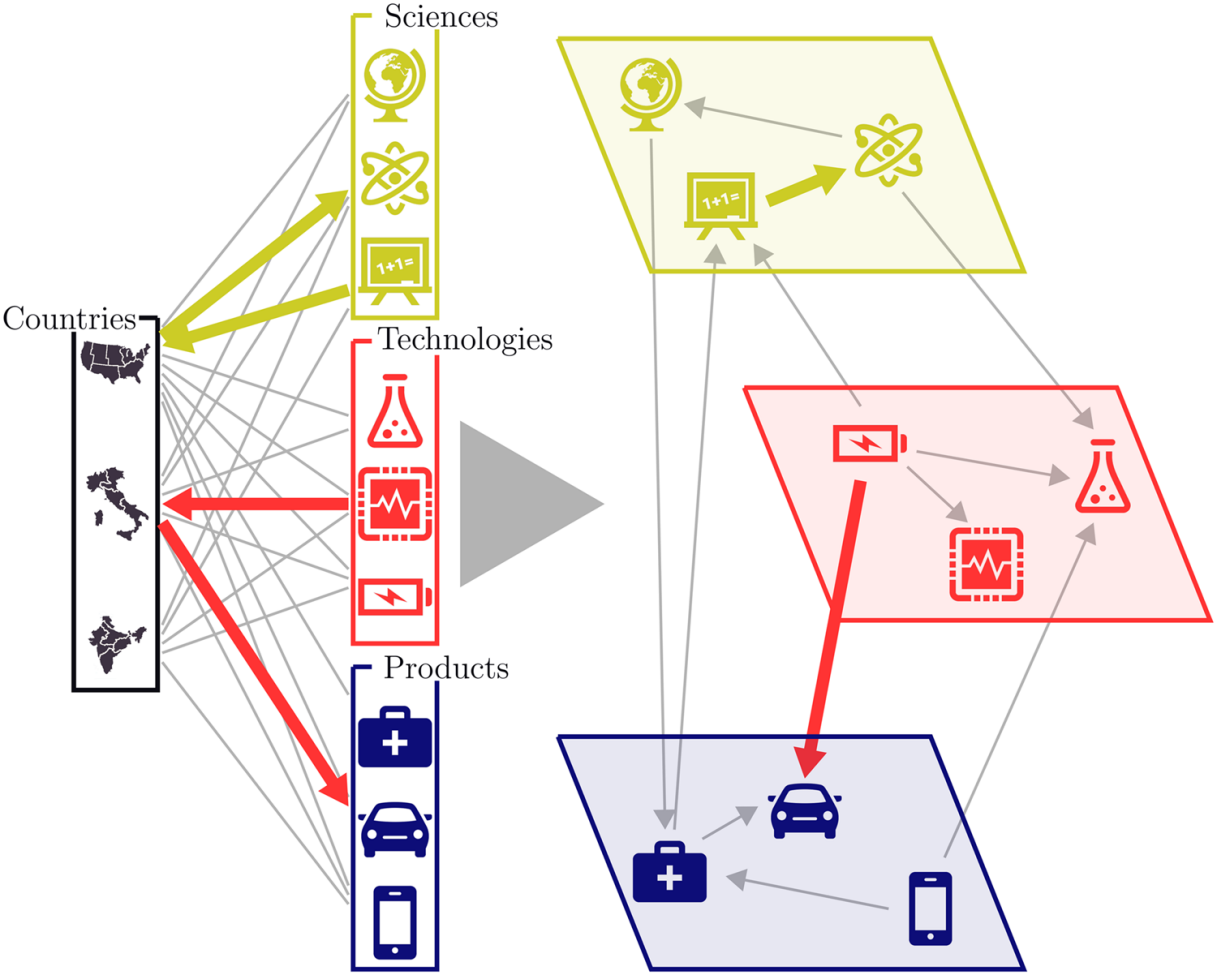
Visual representation of the multilayer space of innovation activities. *Left panel*: Schematic visualization of the triple bipartite network with *C*ountries in one partition and activities (*S*ciences, *T*echnologies, and *P*roducts) in the other one. *Right panel*: Tri-layer representation of the resulting *Assist* matrix between activities. The generic element of the *Assist* matrix is equal to the probability that a bit of information, randomly diffusing in the triple bipartite network, travels from one activity to another. This can happen in the same activity layer, as it is the case for the yellow path linking two sciences, or among different layers, as it is the case for the red path going from a technology to a product.

Using these matrices we compute the probability of having a comparative advantage in activity *a*_2_ ∈ *L*_2_ in the year *y*_2_, conditional to having a comparative advantage in activity *a*_1_ ∈ *L*_1_ in the year *y*_1_ (Fig. 1). This is defined by the *Assist* matrix^17^ (see Methods)

1 $$\begin{array}{rcl}{B}_{{a}_{1}\to {a}_{2}}^{{L}_{1}\to {L}_{2}}({y}_{1},{y}_{2}) & = & Pr({a}_{2};{y}_{2}|{a}_{1};{y}_{1})\\  & = & \sum _{c}\,Pr({a}_{2};{y}_{2}|c,{a}_{1};{y}_{1})Pr(c|{a}_{1};{y}_{1})\\  & = & \sum _{c}\frac{{M}_{c,{a}_{2}}^{{L}_{2}}({y}_{2})}{{d}_{c}^{{L}_{2}}({y}_{2})}\frac{{M}_{c,{a}_{1}}^{{L}_{1}}({y}_{1})}{{u}_{{a}_{1}}^{{L}_{1}}({y}_{1})}\end{array}$$

where ${u}_{{a}_{1}}^{{L}_{1}}({y}_{1})={\sum }_{c}{M}_{c,{a}_{1}}^{{L}_{1}}({y}_{1})$ is the ubiquity of activity *a*_1_ ∈ *L*_1_ in year *y*_1_, and ${d}_{c}^{{L}_{2}}({y}_{2})={\sum }_{{a}_{2}\in {L}_{2}}{M}_{c,{a}_{2}}^{{L}_{2}}({y}_{2})$ is the diversification of country *c* in the layer *L*_2_ in year *y*_2_. These probabilities can be associated with the overlap between the capabilities required to perform activities *a*_1_ in the year *y*_1_ and *a*_2_ in the year *y*_2_, under the assumption that these capability requirements are the same for each country. However we are not assuming that capabilities of individual countries in different years are independent: indeed, these dependencies are intrinsically present in the data we use.

In order to interpret the empirical observed values of ${B}_{{a}_{1}\to {a}_{2}}^{{L}_{1}\to {L}_{2}}({y}_{1},{y}_{2})$, we have to assess their statistical significance using a null model based on appropriate network randomization techniques^22–25^. This model is built on the assumption that the ubiquity of activities and diversification of countries in each layer sum up all the information. The corresponding null hypothesis is thus that activities are independent, and there is no capability structure behind the networks: co-occurrences between activities happens at random, some more likely than others only because of the ubiquities of the two activities – *e.g*., some technological fields and the export of some products are less ubiquitous and thus they are both more likely to be performed by advanced and more diversified countries. Therefore, any specific observed link *a*_1_ → *a*_2_ for which we can reject such null hypothesis is interpreted as the signal of some real interdependency between the specific capabilities required by a country to perform those specific activities – either at the same time or with a time delay.

## Results and Discussion

**Figure 2:**
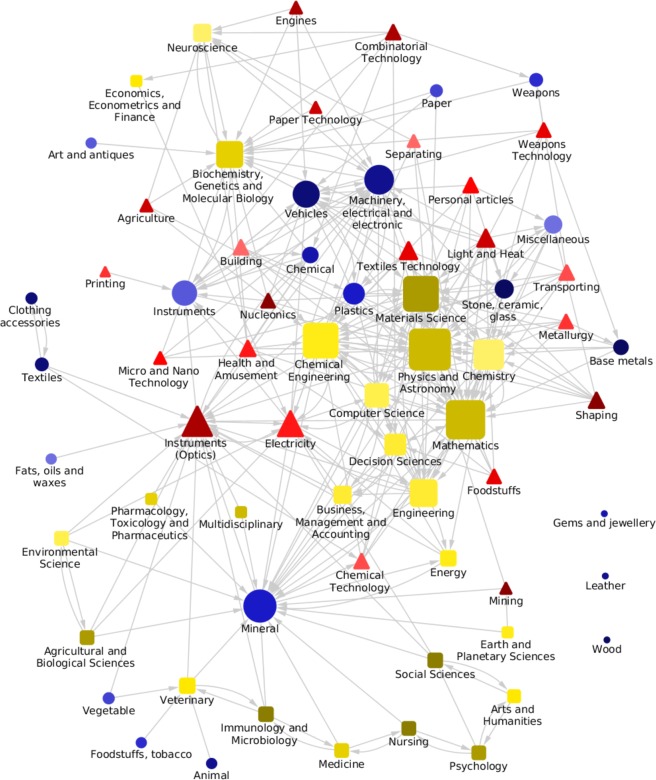
Multilayer network of broadly aggregated activities. The network includes 23 scientific major categories, 25 technological sub-sections, and 21 product sections. Links are obtained using a significance level of 99.999%. To increase the signal-to-noise ratio, we compute *B* as the average of three consecutive years in the middle of our sample (2008–2010). Red triangular nodes represent technologies, yellow squared nodes represent scientific fields and finally blue circle nodes represent the export of products. The node sizes are proportional to their degree.

**Figure 3:**
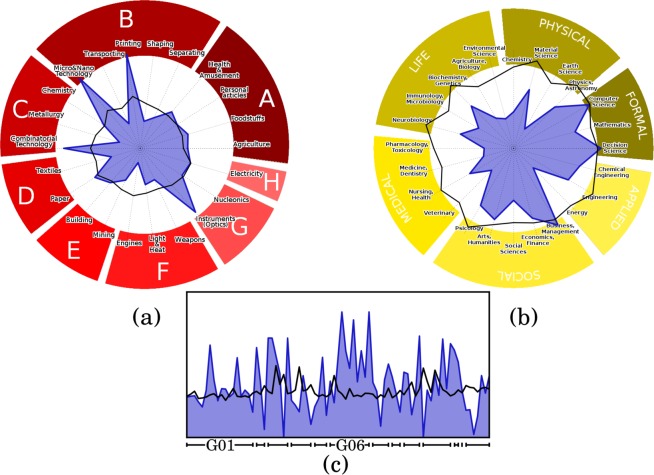
Sample analysis for “export of Desktop Computers” (Harmonized System code 847149) in 2006–2010. The radar plots (**a**,**b**) show how a successful export in Desktop Computers is related to the various technology (**a**) and science (**b**) fields in 2004–2008. The blue contour corresponds to the empirical values of the assist matrix *B*, while the black line denotes the 95% confidence interval under the null model (we do not report the 5% confidence interval here). A technological or scientific field is significantly related to the export of Desktop Computers if the blue silhouette exceeds the confidence interval. We can thus see that while (**a**) shows many fields where co-occurrences are not explained by random noise, (**b**) shows that only a few fields are significantly above the noise, and barely so. Panel (c) reports a higher resolution analysis with technological section “G: Physics” expanded in its classes (three digits codes) and sub-classes (four digits codes) on the horizontal axis. Blue and black lines have the same meaning of panels (a) and (b). The peaks in “G06” corresponds to the “Computing” class.

The described methodology allows obtaining unprecedented qualitative and quantitative insights on the complex dynamics of development. By linking together those activities which are related at a given significance level, we can build the whole multilayer space in which scientific, technological and industrial activities are embedded (Fig. 2). We also perform a more focused analysis and show how a detailed activity (*e.g*., the export of an individual product) is related to activities in other layers at various aggregation levels. An example is shown in Fig. 3, where we plot the scientific and technological fields related to the export of Desktop Computers. We can draw from the figure two observations: (i) significant peaks, *i.e*., values of assist matrix elements observed in the real data with less than 5% probability to occur in the null model, are meaningful according to our understanding of the scientific and technological prerequisites to be competitive in Desktop Computers export; (ii) technology tends to be more significantly related than science to the export of this product, the overall significance of assist matrix entries (*i.e*., the signal-to-noise ratio) being higher in the first case. This is not an exception related to this product, as we shall see next.

The representation of Fig. 2 suggests that any modeling of the Innovation System assuming a standard direction of the dynamics between layers (e.g., from Science to Technology to Products) is simplistic. In other words the assumption that all activities belonging to the same layer behave similarly in determining the innovation cascade is empirically unjustified: the division in scientific, technological and production activities is not greatly informative of their role in the network. A technological activity could be a precursor of some scientific activities, while the opposite could be true for different activities in the same layers: a complex system where no set of labels is fully informative of the dynamic structure. We can however provide some insights on the average interactions between activities in two specific layers to assess if the aggregate models of the innovation system^1^ offer at least a proper description of the most common modes of interaction. Given two layers *L*_1_ and *L*_2_ and a time lag Δ*y*, we compute a signal-to-noise ratio ${\Phi }^{{L}_{1}\to {L}_{2}}(\Delta y)$ as the average for different years *y* of the fraction of significant links *a*_1_ → *a*_2_ in the matrix ${B}_{{a}_{1}\to {a}_{2}}^{{L}_{1}\to {L}_{2}}(y,y+\Delta )$. We consider the links significantly more co-occurring than random with a 99% confidence level. Therefore for two unrelated layers we expect Φ ≃ 1%, and any value above that is the footprint of signal overcoming the noise. Repeating this simple exercise for different temporal windows allows shedding light on the following issues: What is the average influence of the activities in *L*_1_ on the activities in *L*_2_ after a given time? How many years does it take to maximize such impact?

**Figure 4:**
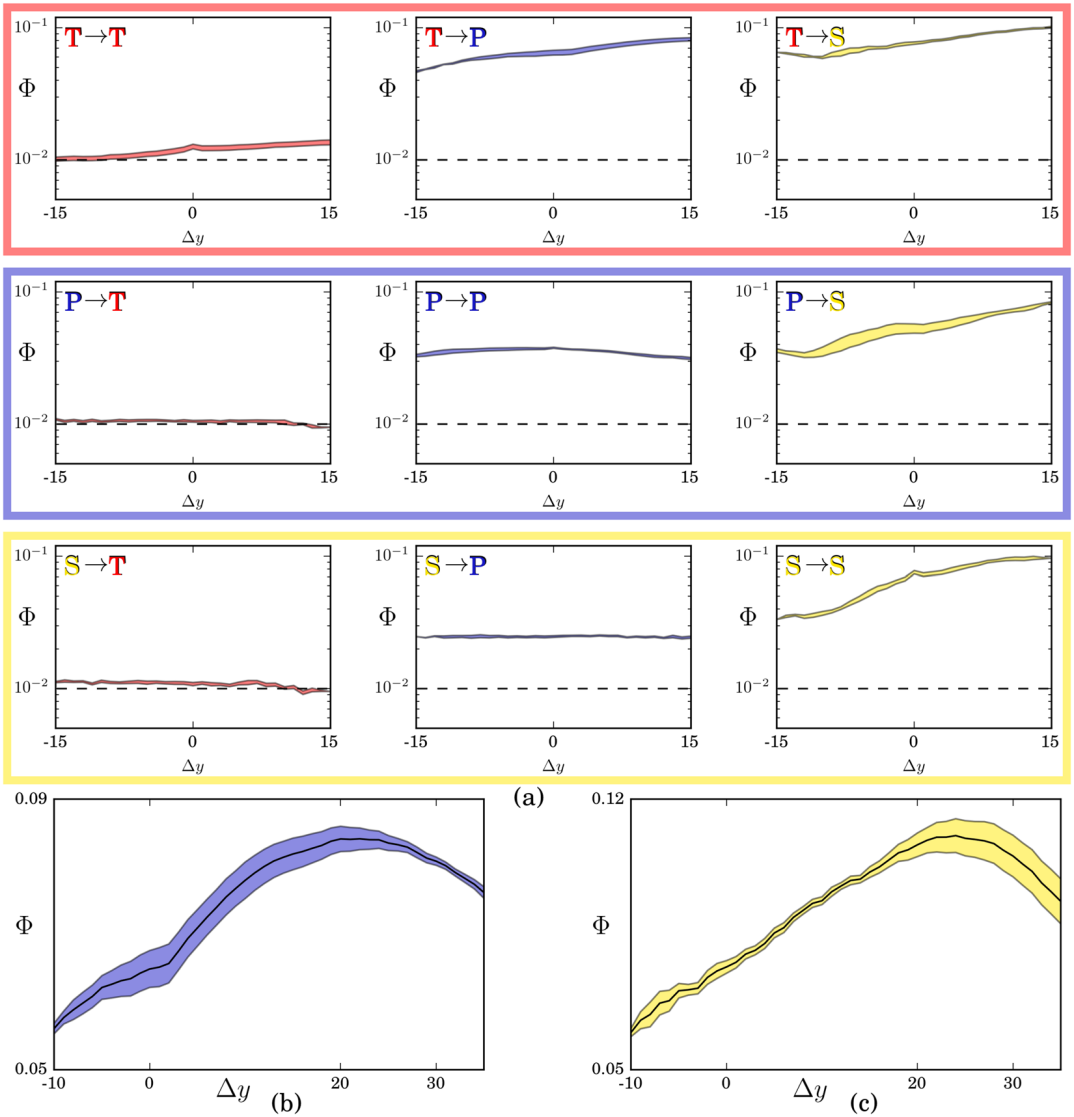
Average interactions between layers for varying time lag Δ*y*. In panel (a), each plot displays the signal ${\Phi }^{{L}_{1}\to {L}_{2}}(\Delta y)$ given by the fraction of significant links going from the activities of layer *L*_1_ to the activities of layer *L*_2_. *L*_1_ varies across rows and *L*_2_ varies across columns, the order being *T*echnology, *P*roducts and *S*cience. The time series are build aggregating three years at a time and looking at all the possible pair of years giving the desired Δ*y*. The shaded area denote the one sigma confidence interval. The analysis is done at a medium level of disaggregation: *T*echnology is split in subclasses (~600), *P*roduction at 4 digits level (~1000) and *S*cience in categories (~300). Dashed black lines mark the noise level Φ = 1%, as we consider significant links at the 99% confidence interval. The same analysis with a longer time frame is reported in panels (b) and (c) respectively for *T*echnology → *P*roducts and *T*echnology → Science relations.

The results, shown in Fig. 4, are striking. First, the signal between different layers is (almost everywhere, see ahead) very high, even for long time differences. Beyond the slowly-changing structure of countries’ activity, this is explained with the strong overlap of countries’ capabilities on different layers. For instance, the ability to patent successfully in a given technological field is a strong predictor for the successful export of specific products and the publication of papers in specific scientific fields. Second, the technology layer is clearly the best precursor for both science and export, and in both cases the signal reaches its maximum after around 20 years: knowing a country’s preferred technologies today gives the highest predictive power for its preferred scientific fields and market sectors in about two decades. It is also the layer more difficult to influence: the capabilities in science and technology in a given year do not give any information on the technological activities in the future. Third, the scientific layer is both the most capability driven, its future activities being strongly related to the present technological, production and even scientific activities, and the least informative about the future activities of the country. Importantly, a higher significance level corresponds to higher accuracy when predicting which activities countries will become proficient with in the future (see the Supplementary Information [Prediction Accuracy] for a recommender systems approach^26^ to this task).

Notice however that we are not giving a direct causal interpretation, saying for instance that there is no impact of science on technology. Rather we measure conditional probabilities for the co-occurrence of activities within the same country at a given time delay. From this viewpoint, having more signal going from present technology to future science than the viceversa is less counterintuive, because the technological spillovers of scientific activities are not appropriable by the economic actors in a country, while patented technologies are. In other words, the chance of someone producing a new patent after reading a scientific paper is not easily appropriable by the country, while possible advances of science due to new technologies (like the discovery of superconductors after advancements in cryogenic techniques) are more likely to be localized in the same country. Moreover, while a technology can have a deep impact on specific scientific fields, scientific research can potentially lead to wide technology spillovers in every fields (think for instance of scientists at CERN inventing the World Wide Web), which however leave no footprint in our signal. Nevertheless we can confidently say that knowing the technological portfolio of a country gives more information on the future scientific fields than the opposite.

Overall, the methods and techniques we presented – in addition to shed light on the dynamics of innovation – can find invaluable use to forecast the scientific fields and market sectors in which countries can have (and will have) a competitive advantage based on their current patent portfolios (see^27^ and the Supplementary Information [Case Study 1 and 2] for instances of implementation of our methodology by international organizations for policy making and applied research). The technical capabilities a country possesses today will define the scientific and market opportunities for the next generation.

## Methods

### Data

The data that support the findings of this study are available from www.scival.com (SciVal), www.epo.org/searching-for-patents/business/patstat (PATSTAT) and https://comtrade.un.org/ (UN COMTRADE), but restrictions apply to the availability of these data, which were used under license for the current study, and so are not publicly available. Processed data on coarse-grained Assist matrices are however available from the authors upon reasonable request.

#### Science

We use data on scientific productivity and impact of countries collected from the SciVal platform, a new API aggregating data from Scopus. The database covers journals, trade publications, book series, conference proceedings, and books. Note that while Scopus was shown to have a broader coverage than Web of Science, and to have a more reliable classification system than Google Scholar, analyses based on either of these databases usually yields very similar results—especially when performed at the country level^28^.

Collected data cover years from 1996 to 2013 and refer to the corpus of scientific publications, each belonging to a given scientific sector (or sub-sector). Data are then aggregated at the country level, so that *W*_*c*,*s*_^*S*^(*y*) is the number of scientific documents produced by country *c* in scientific sector *s* during year *y*. Note that these values are computed using a full counting method for internationally co-authored papers, which may cause a bias towards small countries with high level of internationalization to the detriment of large standalone countries^29^. Biases can arise also for anglophone countries in Social Science and Humanities, as documents written in other languages and published in national journals—which are important especially in such sectors—are not covered in full^30^. However, these sectors are presumingly less connected to patenting and productive activities, and as such do not hinder the results of our analyses.

#### Technology

We use patent data contained in PATSTAT to measure technological capabilities of countries. PATSTAT collects all the patents (approximately 100 millions) by different Patent Offices (almost 100) all around the World. The time span is extremely broad, going from mid-19th century to today. Patent Offices organize knowledge by tagging each patent with one or more codes from the International Patent Classification (IPC). IPC codes define a hierarchical classification consisting of six levels (*sections, sub-sections, classes, sub-classes, groups, sub-groups*), ranging between 8 and over 70 thousand codes (note that we discard classes “99” and sub-classes “Z”, as they represent other technologies not classified in other classes or sub-classes, and they are therefore not well defined). PATSTAT also records the country of origin of the applicant (usually a firm) of each patent. Finally, PATSTAT defines “families” of patents according to primary citations among them^31^, *i.e*., all patents with common priorities. Indeed, multiple patents could be referred to the same innovation, for example because the same firm applied to different patent offices to extend the protection of their patent to wider markets, or because specific patent offices have heterogeneous regulation about the limits of one patent. In particular we use INPADOC families, or extended family, which collects all the documents that are directly or indirectly linked by one or more priorities.

The basic units of observation are thus the families of patents. Each family is related to one or more countries, through the origin of the applicants of the patents in the family, and one or more technological code. Of course the technological codes depend on the aggregation used. In constructing the matrices, we assume that each family counts as a unit and thus weights accordingly within the matrix. Hence, for each family found in our dataset in a given year, we evenly split its unit of weight among all the technology codes and all the countries it maps to, for the years in which there were *applications* for patents in that family. With these caveats in mind, we define *W*_*c*,*t*_^*T*^(*y*) as the number of patent families, or the attributed parts of such families, in the field *t* (indexed by its IPC code) applied (*i.e*., filed, not granted) by firms located in country *c* on year *y*.

#### Products

To proxy economic production we use the BACI export data, recorded by the UN COMTRADE and processed by CEPII^32^. The original database reports the import-export flows among countries with a data span 20 years, from 1995 to 2014. This database includes about 5000 products classified according to the Harmonized System 2007 of the World Customs Organization, which denotes them with a set of 6-digits codes organized in a hierarchical way. A given code is divided into three 2-digit parts, each specifying one level of the hierarchy. Hence, the first part indicates the broadest categories, such as “live animals and animal products” (01xxxx) or “plastics and articles thereof” (39xxxx). The second two digits specify further distinctions in each category, such as “live swine” (0103xx) or “live bovines” (0102xx), while the last two digit are even more specific. The trade flows are quantified in thousands of current US dollars. After a data sanitation procedure, a country-product matrix is obtained, whose generic element *W*_*c*,*p*_^*P*^(*y*) represents the monetary value of the overall export of country *c* for product *p* during year *y*.

Note that by using these data we are implicitly assuming that export is a good proxy for production. Even if this hypothesis is frequent in the economic complexity literature^13,14^, we stress that since we are interested in quantifying the capabilities and the competitiveness of countries, using export themselves could be even better than using data on production. Indeed, the very fact to be able to export competitively a given product is an even clearer signal of having the required capabilities, being instead production biased by country-based effects and internal demand. In this respect, internal demand can represent a capability itself, required to develop a comparative advantage. Note also that UN-COMTRADE data take into account the re-exports of countries and so imports do not need to be taken into considerations.

A final remark on these data is in order. We measure exports according to their value in dollars, a rather different unit than “number of documents” used for *S*cience and *T*echnology. In these two latter cases, however, the output of countries can be assessed using not only such number, but also the backward citations accrued by those documents — which may be close to the notion of “value”. In the Supplementary Information [Robustness Check] we discuss the various pros and cons of this choice with respect to the aims of the present study, and perform a robustness check by showing that our results are qualitatively unchanged by using backward citations instead of number of documents.

### Analytical techniques

#### Revealed comparative advantage

Given the raw matrices {**W**^*L*^(*y*)} for *L* ∈ {*S*, *T*, *P*} and for the different years *y*, described in the section above, the first task is to determine whether a given country *c* shows a comparative advantage in activity *a* (belonging to layer *L*), both with respect to other countries as well as to other activities of the same kind. This is achieved through the *revealed comparative advantage* (*RCA*)^33^, an intensive metric computed as the ratio between the weight of activity *a* in the activity basket of *c* and the weight of activity *a* in the total world activity. As a comparative advantage is revealed if *RCA* > 1, we binarize the raw matrices to obtain new matrices {**M**^*L*^(*y*)}:

2 $${M}_{c,a}^{L}(y)=\{\begin{array}{l}1\,{\rm{if}}\,\frac{{W}_{c,a}^{L}(y)}{{\sum }_{a^{\prime} }\,{W}_{c,a^{\prime} }^{L}(y)}/\frac{{\sum }_{c^{\prime} }\,{W}_{c^{\prime} ,a}^{L}(y)}{{\sum }_{c^{\prime} ,a^{\prime} }\,{W}_{c^{\prime} ,a^{\prime} }^{L}(y)}\ge 1,\\ 0\,{\rm{otherwise}}.\,\end{array}$$

While originally developed in the economic context, the use of the RCA-like metrics is also common in studies of scientific and technological activities (see for instance^34–38^). The use of the same RCA formulation for the different layer here is mainly motivated by having a coherent way to build them. Note that the RCA filter is properly normalized by making quantities related to different countries, activities and years comparable. Take for instance the *S*cience layer: by assessing the number of papers of a country in a given year and a given scientific sector against the overall world count for that year and that sector, we get rid of both temporal variations and sector differences^39^. And when number of citations is used instead of number of papers, the RCA filter also removes the bias towards old papers which had more time to accrue citations than recent ones^40^ (however, the small numbers typical of recent years can lead to less reliable results). The RCA normalization is also particularly useful to weaken the various issues affecting patent data (see below).

Finally note that the RCA, the standard metric used in economic complexity studies, does provide an effective overall measure for how competitive a country is at the global level, but not a specific assessment with respect to each other country to which the exports are directed. Yet taking into account these “local” effects would require introducing additional parameters and arbitrary assumptions. On one hand this could generate uncontrolled statistical biases; on the other hand, increasing the dimensionality of the problem (*i.e*., increasing the number of variables) can increase spurious mixing of data and thus reduce the time-scale of prediction.

#### The multilayer space

Once the binary matrices {**M**^*L*^(*y*)} are defined, we build the multi-layer space connecting productive, technological and scientific activities inspired by the general ideas presented in^16,17^. Given a pair of layers (*L*_1_, *L*_2_) ∈ {*P*, *T*, *S*}, in order to assess whether countries having a comparative advantage in activity *a*_1_ ∈ *L*_1_ in year *y*_1_ are more likely to have an advantage also in activity *a*_2_ ∈ *L*_2_ in year *y*_2_, we have to perform an appropriate contraction of ${{\bf{M}}}^{{L}_{1}}({y}_{1})$ with ${{\bf{M}}}^{{L}_{2}}({y}_{2})$ over the country dimension (*i.e*., the set *C* of countries), and take the element (*a*_1_, *a*_2_). The detailed prescription to build the *Assist* matrix derives from the so called *probabilistic spreading* approach^41^.

Let us consider a bit of information placed on a generic activity *a*_1_ in layer *L*_1_. We aim at describing how this information can spread to activities in layer *L*_2_. As first step, information jumps to countries according to the connection patterns of ${{\bf{M}}}^{{L}_{1}}({y}_{1})$: the transition probability that the bit of information goes from *a*_1_ to a given country *c* is ${\rho }_{{a}_{1}\to c}^{{L}_{1}\to C}({y}_{1})={M}_{c,{a}_{1}}^{{L}_{1}}({y}_{1}){\textstyle /}{{u}_{{a}_{1}}}^{{L}_{1}}({y}_{1})$, where ${u}_{{a}_{1}}^{{L}_{1}}({y}_{1})=\sum _{c\in C}{M}_{c,{a}_{1}}^{{L}_{1}}({y}_{1})$ is the ubiquity (or degree) of *a*_1_ in *L*_1_ for year *y*_1_. We thus assume equal transition probabilities for countries having a comparative advantage in *a*_1_, which is motivated by the maximum uncertainty principle since we do not want to introduce biases in the processes. As second step, information located on countries jumps to activities in layer *L*_2_, now following the connection patters of ${{\bf{M}}}^{{L}_{2}}({y}_{2})$. Again assuming maximum uncertainty, the transition probability that the bit of information goes from *c* to a given activity *a*_2_ in layer *L*_2_ is ${\rho }_{c\to {a}_{2}}^{C\to {L}_{2}}({y}_{2})={M}_{c,{a}_{2}}^{{L}_{2}}({y}_{2}){\textstyle /}{{d}_{c}}^{{L}_{2}}({y}_{2})$, where ${d}_{c}^{{L}_{2}}({y}_{2})={\sum }_{a}{M}_{c,a}^{{L}_{2}}({y}_{2})$ is the diversification (or degree) of country *c* in layer *L*_2_ for year *y*_2_. Putting these two steps together, the probability that the bit of information jumps from activity *a*_1_ ∈ *L*_1_ to activity *a*_2_ ∈ *L*_2_ finally reads:

3 $$\sum _{c\in C}{\rho }_{{a}_{1}\to c}^{{L}_{1}\to C}({y}_{1}){\rho }_{c\to {a}_{2}}^{C\to {L}_{2}}({y}_{2})=\frac{1}{{u}_{{a}_{1}}^{{L}_{1}}({y}_{1})}\sum _{c\in C}\frac{{M}_{c,{a}_{1}}^{{L}_{1}}({y}_{1}){M}_{c,{a}_{2}}^{{L}_{2}}({y}_{2})}{{d}_{c}^{{L}_{2}}({y}_{2})}\equiv {B}_{{a}_{1}\to {a}_{2}}^{{L}_{1}\to {L}_{2}}({y}_{1},{y}_{2}).$$

The above equation defines a bipartite network between layers *L*_1_ and *L*_2_, which can be interpreted as the flow of information from activities in *L*_1_ in year *y*_1_ to activities in *L*_2_ in year *y*_2_ (*i.e*., after a given time). We can interpret the connections of this network as conditional probabilities *Pr*(*a*_2_; *y*_2_|*a*_1_; *y*_1_) according to the following considerations.

- The bit of information (know-how) associated with a generic activity *a*_1_ is transferred to the various activities in *L*_2_ through the countries having a comparative advantage in *a*_1_.
- In order to account for the highly competitive nature of countries’ development dynamics (be it scientific, technological or economic), we can naturally assume that transferring the know-how from *a*_1_ to any activity *a*_2_ is not convenient for all countries. In particular, in order to get a competitive advantage from *a*_1_, a given country *c* active in *a*_1_ will put an effort in such a transfer which at first approximation is inversely proportional to the number of countries having *a*_1_ in their activity baskets. Thus, the probability that country *c* exploits the information provided by *a*_1_ is given by:

4 $$\Pr (c|{a}_{1};{y}_{1})\equiv {\rho }_{{a}_{1}\to c}^{{L}_{1}\to C}({y}_{1}).$$

- When a country *c* transfers its know-how in *a*_1_ to activities in *L*_2_, it is natural to assume that a specific activity *a*_2_ will be chosen with a probability inversely proportional to the number of activities in *L*_2_ in which country *c* is active. This assumption derives from the finite and fixed amount of resources every country is endowed with for activity transfer. Thus, the conditional probability for the transfer of know-how from activity *a*_1_ to activity *a*_2_ performed by a given country *c* reads:

5 $$\begin{array}{rcl}Pr({a}_{2};{y}_{2}|c;{a}_{1};{y}_{1})Pr(c|{a}_{1};{y}_{1}) & \equiv  & [{\rho }_{c\to {a}_{2}}^{C\to {L}_{2}}({y}_{2}){M}_{c,{a}_{1}}^{{L}_{1}}({y}_{1})]{\rho }_{{a}_{1}\to c}^{{L}_{1}\to C}({y}_{1})\\  & = & {\rho }_{c\to {a}_{2}}^{C\to {L}_{2}}({y}_{2}){\rho }_{{a}_{1}\to c}^{{L}_{1}\to C}({y}_{1})\end{array}$$

where the term *Pr*(*a*_2_;*y*_2_|*c*;*a*_1_;*y*_1_) contains the dependence on the starting point through ${M}_{c,a1}^{{L}_{1}}({y}_{1})$, yet the final equality holds since ${[{M}_{c,{a}_{1}}^{{L}_{1}}({y}_{1})]}^{2}={M}_{c,{a}_{1}}^{{L}_{1}}({y}_{1})$.

Finally, the probability composition formula to assess the transition probability from activity *a*_1_ to activity *a*_2_ leads directly to Eq. (3):

6 $${B}_{{a}_{1}\to {a}_{2}}^{{L}_{1}\to {L}_{2}}({y}_{1},{y}_{2})\equiv Pr({a}_{2};{y}_{2}|{a}_{1};{y}_{1})=\sum _{c\in C}Pr({a}_{2};{y}_{2}|c,{a}_{1};{y}_{1})Pr(c|{a}_{1};{y}_{1}).$$

Note that the time direction of the process is given by the time lag *y*_2_ − *y*_1_. In the case *y*_1_ > *y*_2_, Eq. 3 remains unchanged (still representing the transition probability from *a*_1_ to *a*_2_), but the interpretation in terms of information flows is the opposite: ${B}_{{a}_{1}\to {a}_{2}}^{{L}_{1}\to {L}_{2}}({y}_{1},{y}_{2})$ becomes the probability that a bit of information reaching activity *a*_1_ ∈ *L*_1_ originally came from activity *a*_2_ ∈ *L*_2_.

#### Statistical hypothesis testing

To assess the statistical significance of elements of the Assist matrices, we resort to a null model for the bipartite matrices {**M**^*L*^(*y*)}, built by randomly reshuffling their elements (*i.e*., the network connections of layer *L*), but preserving country diversifications and activity ubiquities (*i.e*., degrees). This means that we wipe out the signal coming from the network connectivity patters, beyond that encoded by the degree values. Yet in order to formalize the null model analytically (and thus avoid relying on a conditional uniform graph test^42^), degree constraints are imposed on average—as for the Canonical ensemble in Statistical Mechanics. We thus end up with a null hypothesis described by the *Bipartite Configuration Model* (BiCM)^22^, an extension of the standard *Configuration Model*^43^ to bipartite networks.

Formally, the BiCM null model for a given matrix **M**^*L*^(*y*) is defined as the ensemble Ω^*L*^(*y*) of bipartite network configurations which are maximally random, except for the ensemble average of the degrees that are constrained to the values observed in the empirical network: ${\langle {\tilde{d}}_{c}^{L}(y)\rangle }_{{\Omega }^{L}(y)}={d}_{c}^{L}(y)$ ∀*c* ∈ *C* and ${\langle {\tilde{u}}_{a}^{L}(y)\rangle }_{{\Omega }^{L}(y)}={u}_{a}^{L}(y)$ ∀*a* ∈ *L*. To ease the notation, in the following we omit the explicit dependence of quantities on the layer *L* and year *y*, which do not vary throughout the construction of the BiCM. Furthermore, we use symbols with the tilde for quantities assessed on null model configurations, and without the tilde for observed values.

Let $\tilde{{\bf{M}}}\in \Omega$ be a network configuration in the ensemble and $P(\tilde{{\bf{M}}})$ be the probability of that graph within the ensemble. Following the statistical mechanics prescriptions, the least biased choice of probability distribution is the one that maximizes the Gibbs entropy

7 $$S=-\,\sum _{\tilde{{\bf{M}}}\in \Omega }P(\tilde{{\bf{M}}})\,\mathrm{ln}\,P(\tilde{{\bf{M}}}),$$

subject to the normalization condition $\sum _{\tilde{{\bf{M}}}\in \Omega }P(\tilde{{\bf{M}}})=1$ plus the constraints:

8 $$\begin{array}{rcl}{\langle {\tilde{d}}_{c}\rangle }_{\Omega } & = & \sum _{\tilde{{\bf{M}}}\in \Omega }\,P(\tilde{{\bf{M}}}){\tilde{d}}_{c}(\tilde{{\bf{M}}})={d}_{c}\,\forall \,c\in C,\\ {\langle {\tilde{u}}_{a}\rangle }_{\Omega } & = & \sum _{\tilde{{\bf{M}}}\in \Omega }\,P(\tilde{{\bf{M}}})\,{\tilde{u}}_{a}(\tilde{{\bf{M}}})={u}_{a}\,\forall \,a\in L.\end{array}$$

Introducing the respective Lagrange multipliers *ω*, {*μ*_*c*_}_*c*∈*C*_ and {*ν*_*a*_}_*a*∈*L*_ (one for each country and activity of the network), the probability distribution that maximizes the entropy satisfies, for all configurations $\tilde{{\bf{M}}}\in \Omega$:

9 $$\begin{array}{rcl}0 & = & \frac{\delta }{\delta P(\tilde{{\bf{M}}})}[S+\omega (1-\sum _{\tilde{{\bf{M}}}\in \Omega }P(\tilde{{\bf{M}}}))+\sum _{c\in C}\,{\mu }_{c}({d}_{c}-\sum _{\tilde{{\bf{M}}}\in \Omega }P(\tilde{{\bf{M}}})\,{\tilde{d}}_{c}(\tilde{{\bf{M}}}))\\  &  & +\sum _{a\in L}\,{\nu }_{a}({u}_{a}-\sum _{\tilde{{\bf{M}}}\in \Omega }P{\boldsymbol{(}}\tilde{{\bf{M}}}{\boldsymbol{)}}\,{\tilde{u}}_{a}(\tilde{{\bf{M}}}{\boldsymbol{)}})].\end{array}$$

The solution is:

10 $$P(\tilde{{\bf{M}}}|\{{\mu }_{c}\},\{{\nu }_{a}\})={e}^{-H(\tilde{{\bf{M}}}|\{{\mu }_{c}\},\{{\nu }_{a}\})}/Z(\{{\mu }_{c}\},\{{\nu }_{a}\}),$$

where $H(\tilde{{\bf{M}}}|\{{\mu }_{c}\},\{{\nu }_{a}\})$ is the graph Hamiltonian and *Z*({*μ*_*c*_},{*ν*_*a*_}) is the partition function

11 $$H(\tilde{{\bf{M}}}|\{{\mu }_{c}\},\{{\nu }_{a}\})=\sum _{c\in C}\,{\mu }_{c}\,{\tilde{d}}_{c}(\tilde{{\bf{M}}})+\sum _{a\in L}\,{\nu }_{a}\,{\tilde{u}}_{a}(\tilde{{\bf{M}}}),$$

12 $$Z(\{{\mu }_{c}\},\{{\nu }_{a}\})={e}^{\omega +1}=\sum _{\tilde{{\bf{M}}}\in \Omega }\,{e}^{-H(\tilde{{\bf{M}}}|\{{\mu }_{c}\},\{{\nu }_{a}\})}.$$

Equations (10), (11) and (12) define the BiCM model, namely the distribution over a specified set of network configurations that maximizes the entropy subject to the known constraints. As we are considering local constraints (the degrees), we can work out on Eq. (10) to obtain^22^:

13 $$P(\tilde{{\bf{M}}}|\{{\mu }_{c}\},\{{\nu }_{a}\})=\prod _{c\in C}\,\prod _{a\in L}\,{\pi }_{c,a}^{{\tilde{M}}_{c,a}}\,{(1-{\pi }_{c,a})}^{{\tilde{M}}_{c,a}},$$

where *π*_*c*,*a*_ is the ensemble probability for the connection between country *c* and activity *a*:

14 $${\pi }_{c,a}={\langle {\tilde{M}}_{c,a}\rangle }_{\Omega }=\sum _{\tilde{{\bf{M}}}\in \Omega }\,{\tilde{M}}_{c,a}\,P(\tilde{{\bf{M}}}\,|\{{\mu }_{c}\},\{{\nu }_{a}\})=\frac{{\eta }_{c}{\theta }_{a}}{1+{\eta }_{c}{\theta }_{a}}$$

with *η*_*c*_ = *e*^−*μ*^_*c*_ and *θ*_*a*_ = *e*^−*ν*^_*a*_. Note that in Eq. (13) the network probability is obtained as the product of connection probabilities over all possible country-activity pairs, meaning that in the BiCM context links results as independent random variables. The probability distribution in Eq. (13) yet depends on the values of the Lagrange multipliers, which have to be estimated as:

15 $$\begin{array}{rcl}-\frac{\partial }{\partial {\mu }_{c}}\,\mathrm{ln}\,Z(\{{\mu }_{c}\},\{{\nu }_{a}\}) & = & {\langle {\tilde{d}}_{c}\rangle }_{\Omega }\,\forall \,c\in C\\ -\frac{\partial }{\partial {\nu }_{a}}\,\mathrm{ln}\,Z(\{{\mu }_{c}\},\{{\nu }_{a}\}) & = & {\langle {\tilde{u}}_{a}\rangle }_{\Omega }\,\forall \,a\in L.\end{array}$$

To obtain the numerical value of the ensemble average of the constraints, we maximize the log-likelihood function:

16 $$\begin{array}{c} {\mathcal L} (\{{\mu }_{c}\},\{{\nu }_{a}\})=\,\mathrm{ln}\,P({\bf{M}}|\{{\mu }_{c}\},\{{\nu }_{a}\})=\sum _{c\in C}\,{d}_{c}\,\mathrm{ln}\,{\eta }_{c}\\ \,\,\,\,\,\,+\,\sum _{a\in L}\,{u}_{a}\,\mathrm{ln}\,{\theta }_{a}-\sum _{c\in C}\,\sum _{a\in L}\,\mathrm{ln}(1+{\eta }_{c}\,{\theta }_{a}),\end{array}$$

which means solving the system of |*C*| + |*L*| equations in |*C*| + |*L*| unknowns:

17 $$\{\begin{array}{c}{d}_{c}=\sum _{a\in L}\,{\pi }_{c,a}=\sum _{a\in L}\,\frac{{\eta }_{c}\,{\theta }_{a}}{1+{\eta }_{c}\,{\theta }_{a}}\,\forall \,c\in C\\ {u}_{a}=\sum _{c\in C}\,{\pi }_{c,a}=\sum _{c\in C}\,\frac{{\eta }_{c}\,{\theta }_{a}}{1+{\eta }_{c}\,{\theta }_{a}}\,\forall \,a\in L\end{array}$$

Connection probabilities of Eq. (14) are now well defined, and can be used to directly sample the ensemble of bipartite configurations or to compute the quantities of interest analytically.

Reintroducing in the notation the explicit dependence on the layer *L* and year *y*, we finally build the null model ${\Omega }^{{L}_{1}\to {L}_{2}}({y}_{1},{y}_{2})$ for the Assist matrix from layer *L*_1_ at *y*_1_ to layer *L*_2_ at *y*_2_. This is done by contracting the two BiCMs for the matrices ${{\bf{M}}}^{{L}_{1}}({y}_{1})$ and ${{\bf{M}}}^{{L}_{2}}({y}_{2})$ along the country dimension, as for Eq. (3). We have:

18 $${\tilde{B}}_{{a}_{1}\to {a}_{2}}^{{L}_{1}\to {L}_{2}}({y}_{1},{y}_{2})=\frac{1}{{\tilde{u}}_{{a}_{1}}^{{L}_{1}}({y}_{1})}\sum _{c\in C}\,\frac{{\tilde{M}}_{c,{a}_{1}}^{{L}_{1}}({y}_{1}){\tilde{M}}_{c,{a}_{2}}^{{L}_{2}}({y}_{2})}{{\tilde{d}}_{c}^{{L}_{2}}({y}_{2})}.$$

The distributions of ${\tilde{B}}_{{a}_{1}\to {a}_{2}}^{{L}_{1}\to {L}_{2}}({y}_{1},{y}_{2})$ values describing the null model can be in principle obtained using exact techniques^23,24^. Due to the non-Gaussianity of such distributions, here we resort to a more practical sampling technique. We thus use Eqs. (14) and (18) to generate null Assist matrix networks, and populate the ensemble ${\Omega }^{{L}_{1}\to {L}_{2}}({y}_{1},{y}_{2})$ to estimate the full distributions. The generic observed element ${B}_{{a}_{1}\to {a}_{2}}^{{L}_{1}\to {L}_{2}}({y}_{1},{y}_{2})$ is then deemed statistically significant depending on the *p*-value that we can infer from its distribution under the null hypothesis. The specific threshold for statistical significance and the size of the generated ensemble vary on the exercises performed (as highlighted in the text). It is useful to recall that the two choices, the threshold and the size of the ensemble, are not unrelated: the higher the threshold we want to test, the bigger the sample we require. At the very least, if we set a 99% threshold we naturally need at least 100 realizations of the null model to distinguish empirical points overcoming the threshold, whereas, at least 1000 realizations would be required to test against a 99.9% threshold. While this bare minimum is not enough if we want to test a specific link, it could be enough when, as in Fig. 1 of the main text, we just want to check the share of significant links out of a large sample. This is indeed the case of the last exercise, whose results are reported in Fig. 4 of the main text. There we fix a pair of layers (*L*_1_, *L*_2_) and a given aggregation level. Then, for a given time lag Δ*y*, we set a measure of signal-to-noise ratio ${\Phi }^{{L}_{1}\to {L}_{2}}(\Delta y)$ equal to average percentage, over the different years *y*, of significant connections observed in the matrix ${{\bf{B}}}^{{L}_{1}\to {L}_{2}}(y,y+\Delta y)$ using a threshold of 95%. Therefore, we expect ${\Phi }^{{L}_{1}\to {L}_{2}}(\Delta y)\simeq 5 \%$ when no signal is found.

#### Scale of analysis

The scale of the analysis (*i.e*., the aggregation level of data) is crucial when performing the various exercises. Indeed, even if we do not use an explicit notation, any analysis can be performed at different aggregation levels, in multiple dimensions. *First*, trivially, we can perform the analysis at different aggregations along the different activities. At a very broad aggregation we can consider “Physics” as one scientific activity, while at a finer aggregation we can consider any subfield of Physics as an activity. *Second*, we can compute co-occurrences at different geographical aggregations: we can look if two activities co-occur in the same countries, or in the same regions. *Third*, we can look at different temporal aggregations: we can compute the raw matrices **W**^*T*^(*y*) looking at the patents produced in one month, in one year or in five years. The choice of the scale of observation can be a relevant ingredient to look at specific effects: for example the capabilities required at the country level to perform an activity, like “diffused security and education”, can be widely different from the capabilities required at the local level, like “infrastructure” or a specific “climatic condition”. In other situations, the choice of the scale of observation can be driven mostly by our specific interest in a more or less granular result. This is for example shown in Fig. 3 of the main text, where we look at the technological fields required to kick-start the export of Desktop Computers at different technological resolutions.

There are however practical reasons constraining the possible resolutions which can be accessed. Data availability is indeed a critical issue. For example, while patents can be assigned to a region at any different scale, both by looking at the address of the inventors and at the address of the assignee firm, exports are recorded by customs and are therefore not easily available at finer geographical resolutions. In general, if we are looking at the same time at scientific papers, patents, and exports, the common geographical resolution cannot be finer than the country, and the time interval cannot be shorter than one year. The second constraining reason is the statistical power of our tests. The finer the activity or time resolution, the less the signal to noise ratio is required to validate each link. This is due to the fact that there will be less activities of a specific kind in a short time, and therefore randomness can play an important role. We have therefore a trade-off, a sort of indetermination principle: if we are interested at specific activities, we have to increase the time window by pooling different years. This can be done both by summing up the matrices for the years in the time interval, or by stacking the matrices by considering different yearly observations of the same country as different rows, as in^17^. This is what we do in the paper when we say that we pool different years. Otherwise, when we are interested in a finer time analysis, like that generating Fig. 4 of the main document, either we look at very aggregated fields or, like we do in that analysis, we ignore the specific fields and we simply look at the *number* of significant fields.

### Typical issues with the use of patents data

There are several issues about measuring technological outputs of countries by patents counting. First, in different fields there is a very different propensity to patenting, and such propensity can vary in time following technological or institutional changes^44^. Second, different countries have different institutional frameworks providing heterogeneous incentives to patenting. Third, partially related to the previous two points, patenting can have different purposes (*e.g*., defensive patenting) other than the protection of a specific output business-related R&D. Fourth, not all technology is patented, and in particular patent holders may also expect future returns, therefore a technological indicator based on patents can have a natural relation with industrial productivity. These issues are common to the literature on patents, and are actually less problematic in our case – since we are using the RCA binary filter (indicating *whether* a country *c* reveals a competitive advantage in field *t* in year *y*), and not actual values. Indeed, by computing the RCA each field specific, country specific, time specific, field-time specific and country-time specific possible bias is washed away by the RCA measure itself. There are still possible biases related to country-field specific effects (*e.g*., regulations are stricter in Europe than US in patents related to ICT) that have to be acknowledged as possible limitations.

A further issue is related to the limited presence of patents in some parts of the technological spectrum. Other than the already discussed sector-specific potential biases, there are biases related to the specific kind of innovation and to the motives behind innovation. For example, due to the monetary cost and monetary reward of patents, patenting is focused on innovations with a strictly economic advantage, *i.e*., innovations for which the inventors can extract an important part of the value added of it. This is in sharp contrast, for example, with scientific research, where different motives (prestige, but also interest and curiosity) lead the choices of actors. In the light of this issue, we could expect that patent data can have a high forecasting power when considering production and export, but could fail to properly forecast future scientific progress. The strong signal observed in the determination of future scientific fields is however reassuring. If anything, had we additional data other than patents at our disposal to represent the technology output of countries, and to properly measure non economically-viable innovation, we could have expected an even higher signal from technology to science.

## Supplementary information

Supplementary Material
