# Supplementary material for: Unfolding the innovation system for the development of countries: coevolution of Science, Technology and Production
Source: Sci Rep. 2019 Nov 11;9:16440. doi: 10.1038/s41598-019-52767-5 (PMC6848202; doi:10.1038/s41598-019-52767-5)
Supplement: Supplementary file 1 — Supplementary Material [file 41598_2019_52767_MOESM1_ESM.pdf]

Unfolding the innovation system for the development of countries: coevolution of Science, Technology and Production

Emanuele Pugliese[1,2,3,†], Giulio Cimini[4,1,*], Aurelio Patelli[1,‡], Andrea Zaccaria[1,2], Luciano Pietronero[5,1,2], Andrea Gabrielli[1,⋆]

[1] Istituto dei Sistemi Complessi (ISC)-CNR - 00185 Rome (Italy)
[2] International Finance Corporation, World Bank Group - 20433 Washington (USA)
[3] University of Bath - Bath BA27AY (United Kingdom)
[4] IMT School for Advanced Studies - 55100 Lucca (Italy)
[5] Dipartimento di Fisica, Sapienza Università di Roma - 00185 Rome (Italy)
[*] corresponding author: giulio.cimini@roma1.infn.it
[†] current address: European Commission, Joint Research Centre (JRC) - 41092 Seville (Spain)
[‡] current address: Service de Physique de l'Etat Condensé, CEA-Saclay - 91191 Gif-sur-Yvette (France)
[⋆] current address: Dipartimento di Ingegneria, Università Roma 3 - 00146 Rome (Italy)

SUPPLEMENTARY MATERIAL

CASE STUDY 1

http://www.lucianopietronero.it/wp-content/uploads/2016/10/SI_case1.pdf

CASE STUDY 2: The Spillovers of Astrophysics

UNITED NATIONS
Office for Outer Space Affairs

agenzia spaziale italiana

This case study comes from the interest of the Italian Space Agency (ASI). ASI recently proposed the Open Universe initiative, whose aim is to promote the provision and the usability of space science data, in collaboration with the United Nations Office for Outer Space Affairs (UNOOSA). ASI and UNOOSA organized an Expert Meeting at ASI headquarters in Rome, by invitation only, in which one of the authors presented an application of the methodologies presented in this article. In particular, ASI kindly requested to study the possible economical, cultural, and scientific consequences of the Open Universe initiative. Our approach is particularly suitable to tackle issues like this one, since any scientific or technological field, and any industrial sector, can be seen as a source of time-delayed increasing for the comparative advantages of other fields or sectors. Two highly correlated scientific fields (Astronomy and Astrophysics, Scopus code 3103, and Space and Planetary Science, Scopus code 1912) were analyzed. We present the results in Figure S1, in which we analyze the time-delayed correlation from Astronomy and Astrophysics and all technological fields, at the level of sections. One can notice that, on average, a country characterized by a fruitful scientific activity in Astrophysics will later patent in the “Separating” and ”Instruments (optics)“ sectors. One can also analyze the technological sub-sectors, finding non trivial results such as Earth Drilling (E21B), Chemical and physical processes, *e.g.*, catalysis (B01JC), and Data processing systems (G06Q).

Such an approach can be very useful also in the context of the submission of scientific projects for national and international calls, where usually an analysis of the possible technological and economical spillovers of the proposed research is highly recommended.

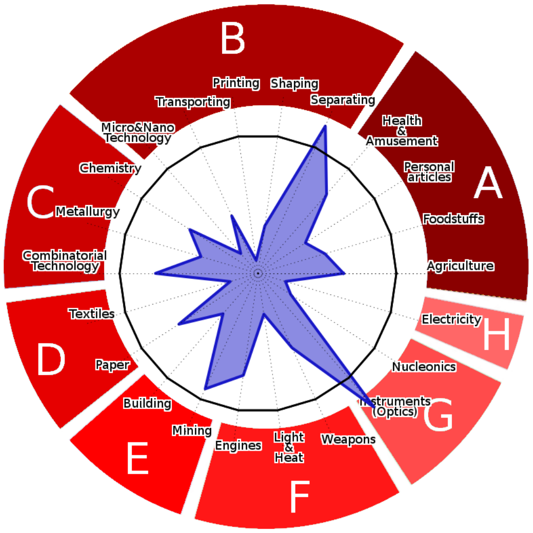

FIG. S1. The radar plot representing the technological fields that will benefit from a revealed comparative advantage in Astronomy and Astrophysics. Two sectors are significant from a statistical point of view: Separating and Instruments (Optics).

ROBUSTNESS CHECK: Citations vs Documents

In the main paper we measure technological and scientific production using the number of documents produced, respectively patents and scientific publications. There are however both empirical and theoretical arguments in favor of using backward citations instead of number of documents. First of all, scientific publications and patents have a very different "values" in different countries and different fields, and institutional difference could affect the number of documents produced. Instead citations are an organic part of the international network, are are thus more robust to this kind of biases. For instance, local incentives could lead a scientist to publish more papers, but it is unlikely to convince the international community to cite this scientist more. Second, since we measure exports in dollars (a measure of value and not a measure of quantity), it would be more natural to use an analogous measure of scientific and technological value, *i.e.*, backward citations, instead of a measure of scientific and technological "quantity", *i.e.*, documents.

There is however a twofold reason for our use of documents, related to the fact that the use of citations requires two points in time: the publication date of the cited document and the publication date of the citing document. First of all, since we are interested in the identification of time lags, the presence of two dates characterizing the same scientific and technological output would force us to make a series of assumptions. Are citations typically done after 3-years or 5-years? Is this time interval the same for scientific publications and patents, and within the different fields? We believe this would add a further layer of noise. Second, since with this work we aim at providing direct policy advices, we must use the most recent data. Take for instance the case of patents: a 5-years backward-citation measure, on top of the time required for patent publication and registration in the database, would generate a time delay of 10 years for data on technologies. This would mean that today we would be using technological data prior to most advances in cloud-computing, smart phones, self driving cars, and so on: a different technological world.

Given these observations, here we want to make clear that our main results are not affected by the use of documents or citations. To this end, we replicate the exercise leading to Figure 4 of the main paper using backward citations for patents and scientific publications instead of number of documents. We thus define the matrices with the number of citations obtained by documents produced by country c in field f and year y, and apply the RCA filter to binarize them. Everything else is left unchanged. Results are reported in Figures S2 and S3. While we observe a slightly smaller signal using citations (probably because of the fuzzier definition of time when citations are used), the general shape and time structure of the signal is invariant: there is no qualitative difference between the use of citations or documents, and overall the reader can read the paper substituting documents for citations if she prefers.

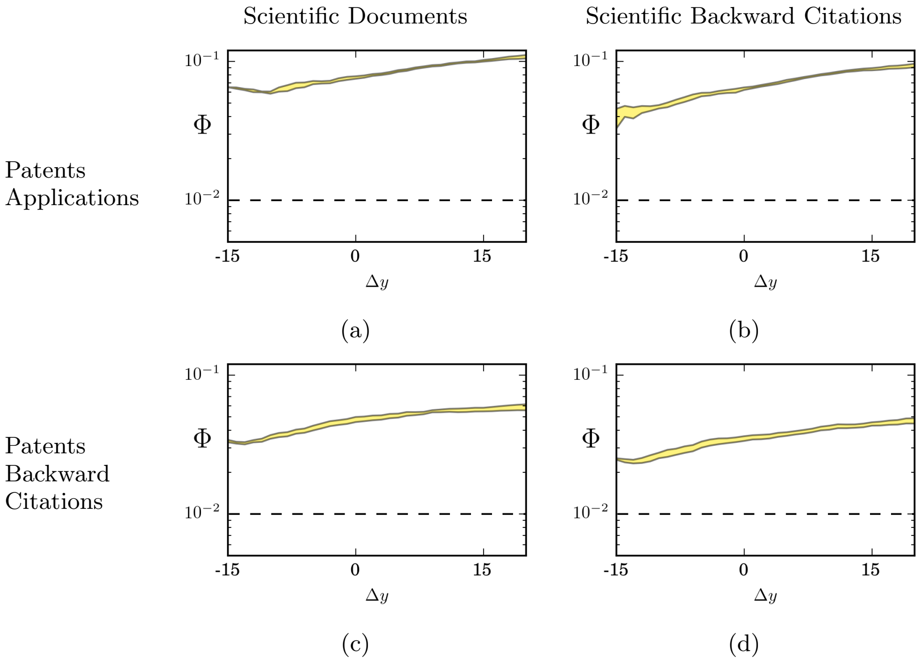

FIG. S2. Share of links going from a technological to a scientific field at time t and $t + \Delta t$ that are significant, with respect to Δt. The different sub-figures reflects the use of documents or citations, sub-figure (a) being the same as the corresponding sub-figure of figure 4 in the main text. While different specifications causes a well defined shift in the share of significant links, the trend is clearly the same.

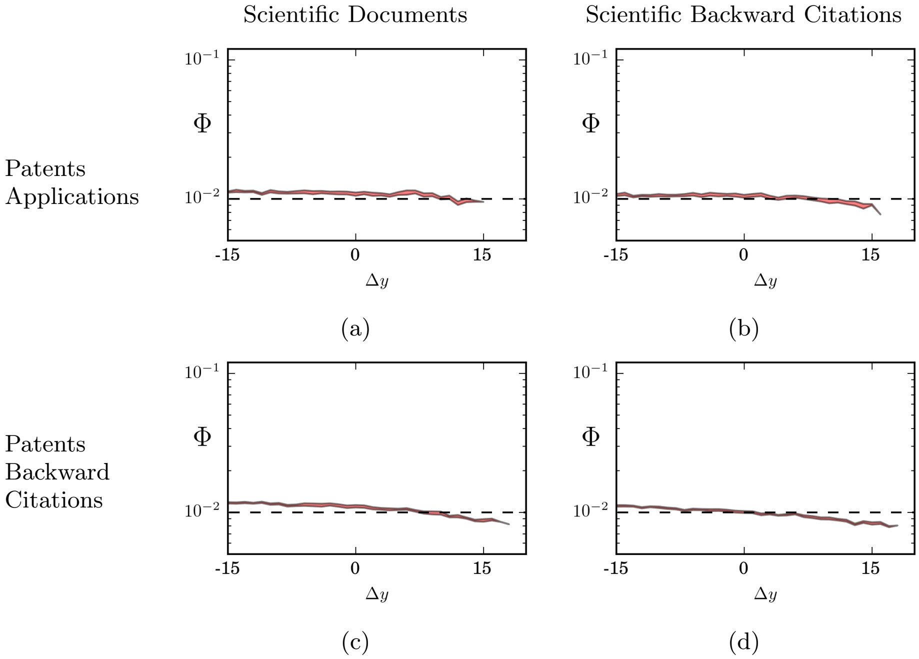

FIG. S3. Share of links going from a scientific to a technological field at time t and $t + \Delta t$ that are significant, with respect to Δt. The different sub-figures reflects the use of documents or citations, sub-figure (a) being the same as the corresponding sub-figure of figure 4 in the main text. In any case there is virtually no signal.

PREDICTION ACCURACY:
The Multilayer Network as a Recommender System

The links of the multilayer network (*i.e.*, the elements of the Assist matrix defined in eq. (1) of the main text) represent probabilities to be competitive in a given activity, conditional to being competitive in another activity, possibly with a time delay. As such, they can be used to predict which countries will become competitive in a given activity after a certain number of years. Here we test the predictive power of our framework using a recommender system approach.

Our aim is to use the information available today (year y_1) to predict whether a link between a country c and a target activity a' will be present in the future (year $y_2 > y_1$). To do so, we assign a *score* σ to each possible link $c - a'$ using the multilayer network computed for years (y_0, y_1) with $y_0 < y_1$. The score is given by the sum of the weights of the links of this network that start from any (*source*) activity a in which the country is competitive at y_1, and end to the activity a' that we want to predict. In formulas:

$$\sigma_{ca'}(y_2) = \sum_a M_{ca}(y_1) B^{L \to L'}_{a \to a'}(y_0, y_1)$$

where $\mathbf{M}(y_1)$ is the source bipartite network activity a belongs to, and $\mathbf{B}(y_0, y_1)$ is the network connecting the source to the target activities. σ is thus our guess of the likelihood that country c will be competitive in a' in the future.

To assess the quality of our recommendations we employ two established performance metrics, *ROC AUC* (the area under the ROC curve) and the *Best F1 score*. *ROC AUC* is an assessment of the overall prediction accuracy that takes into account both the true and the false positive rates. The *F1 score* is the harmonic mean of precision and recall, respectively defined as the fraction of recommended elements that are true positives and the fraction of total positives that are also true. Since both precision and recall depend on the recommendation threshold used to select which scores are associated to the positive and the negative outcomes (*i.e.*, how many items are actually "recommended"), one usually considers the maximum value of this harmonic mean, namely the "best" F1 score.

In the exercise we report here we take data at the same aggregation level used for Figure 4 of the main paper. We then take 2003 as the starting year y_1, build the Assist matrix by considering the average contribution of the years 1998-2000 as y_0 and 2001-2003 as y_1, and use a significance level of 0.01 to validate the links of the Assist matrix. Finally we compare the resulting σ scores with the actual countries activities in the years 2004-2005 (y_2). We compute the goodness of predictions for the three layers separately to quantitatively understand the global influence of the individual layers on themselves and on the other layers. That is, we fix a *source layer* L by limiting the sum in the definition of σ to activities $a \in L$, and a *target layer* L' by computing the recommendation performance indicators considering only activities $a' \in L'$.

Results of this exercise are shown in Figure S4 in a matricial form, where rows represent the source layer L and columns the target layer L'. These results do confirm the findings reported in the main paper. Note that the diagonal elements, which quantify how much a layer is able to predict itself, are the largest entries once the target layer is fixed. Technology has more or less the same predictive power than production with respect to future science, while science has a low predictive power but for science itself. Technology predicts industrial production better than vice-versa.

As a final remark, we note that this exercise can be further extended by considering different performance indicators, different thresholds and different time intervals. For instance, we have replicated the exercise illustrated above using different significance levels for p-values. The outcome is that different performance metrics are maximal for threshold levels that depend on both source and target layers, and of course on the level of data aggregation. This calls for further investigations that will be the object of future work.

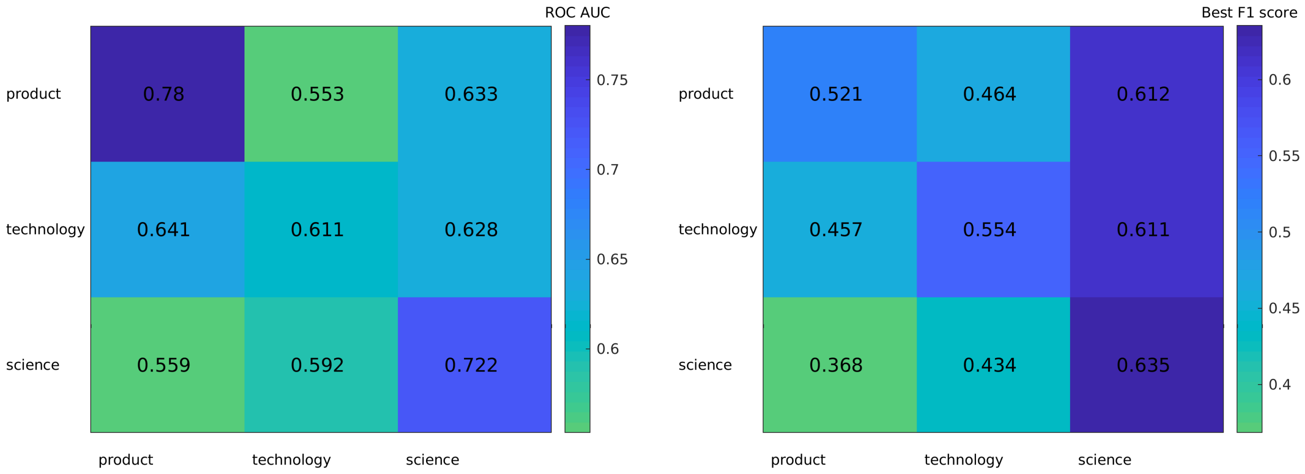

FIG. S4. Results of the recommender system exercise. We use two performance indicators to quantify the ability of the source layer L (rows) to predict the future activities of countries in the target layer L' (columns) after 3 years: the area under the ROC curve (left) and best F1 score (right). Note that for the ROC AUC, a value of 0.5 corresponds to a random classifier, whereas, a value of 1 to a perfect one.
